# Treatment of adult patient with hyperdivergent retrognathic phenotype and anterior open bite: report of a case with non-surgical orthodontic approach

**DOI:** 10.1590/2177-6709.25.4.075-084.bbo

**Published:** 2020

**Authors:** Marinho Del Santo

**Affiliations:** 1 Orthodontist, private practice (São Paulo/SP, Brazil). American Board of Orthodontics certified. Board Brasileiro de Ortodontia certified.

**Keywords:** Hyperdivergent retrognathic phenotype, Anterior open bite, Premolar extractions, Non-surgical orthodontic therapy

## Abstract

Adult patients with anterior open bite and hyperdivergent retrognathic phenotype demand complex treatments, as premolar extractions, molar intrusion or orthognathic surgery. In the present clinical case, a young adult patient without significant growth, with Class I and anterior open bite, was treated with four premolar extractions. The therapeutic result shows good intercuspation, good facial esthetic, good function balance, and stability in a two-year post-fixed treatment follow-up.

## RESUMO

Pacientes adultos com mordida aberta anterior e fenótipo hiperdivergente retrognata demandam tratamentos complexos, com extrações de pré-molares, intrusão de molares ou cirurgia ortognática. No presente caso clínico, uma jovem adulta sem potencial significativo de crescimento, apresentando Classe I e mordida aberta anterior, foi tratada com extrações de quatro primeiros pré-molares. O resultado terapêutico alcançou boa intercuspidação, boa estética facial e bom equilíbrio funcional, e mostrou-se estável depois de dois anos da remoção do aparelho fixo.

## INTRODUCTION

Patients presenting hyperdivergent retrognathic phenotype demand complex orthodontic treatments.^1^ ^,^ ^2^ Etiologically, such phenotype mainly combines the vertical facial genotype with an inadequate mandibular posture.^1^ ^,^ ^3^ Such patients present three mandatory morphologic-functional features: a) deficient ratio between posterior and anterior facial heights, provoking a long and convex facial profile;^4^ ^,^ ^5^ b) deficient masticatory function, with weaker bite force when compared to normal and hypodivergent subjects^6^ ^-^ ^8^, and c) narrower dental arches, especially the maxillary one, with tendency of posterior crossbite occurrence.

Oral breathing is another environmental factor involved in the development of facial hyperdivergence, which evidence of cause-effect has been presented in primates.^9^ Facial hyperdivergence has been related to clinical scenarios as enlarged adenoids,^10^ ^-^ ^14^ allergic rhinitis,^15^ ^,^ ^16^ enlarged tonsils,^17^ and obstructive sleep apnea.^18^ Eating habits and consequently muscle strength are environmental factors also related to facial hyperdivergence.^19^ ^,^ ^20^ In such subjects, it has been postulated that vertical dimensions and mandibular morphology are already established at 6 years of age.^21^ The maxilla presents excessive dentoalveolar growth in the posterior region. Mandibular rami are shorter than in normal and hypodivergent subjects, gonial angles are greater, dentoalveolar growth is excessive in the posterior region as well, the mandibular symphysis is taller and thinner, anterior lower facial height is increased and the mandibular plane angle is steeper.^1^ Such features are associated with clockwise true mandibular rotation, and lesser chin anterior projection.^1^ Transversally, hyperdivergent subjects present narrower dental arches, especially the maxillary, when compared to normal and hypodivergent subjects.^22^ ^-^ ^24^ True mandibular rotation is frequently camouflaged by mandibular remodeling, and only apparent rotation^25^ ^,^ ^26^ is clinically detected by orthodontists.

Contrary to common sense, evidence that support the relationship between anterior open bite and this facial phenotype is weak, mainly because anterior open bite is clearly more dentoalveolar than skeletal.^27^ ^-^ ^29^ However, anterior open bite is a common feature of these subjects, as can be noticed in the present case report.

Many therapeutic protocols have been presented for hyperdivergent retrognathic patients, for example: high-pull headgears,^30^ dental extractions,^31^ ^-^ ^35^ posterior bite-blocks and vertical-pull chincup,^36^ ^-^ ^38^ and orthodontic-surgical approaches.^39^ In the same direction, Buschang et al^40^ showed consistent results pursuing molars intrusion. They described intrusion of upper molars and secondary intrusion (actual or relative) of lower molars, with the use of coil springs and miniscrew implants.^40^

## CASE REPORT

**Figure 1:**
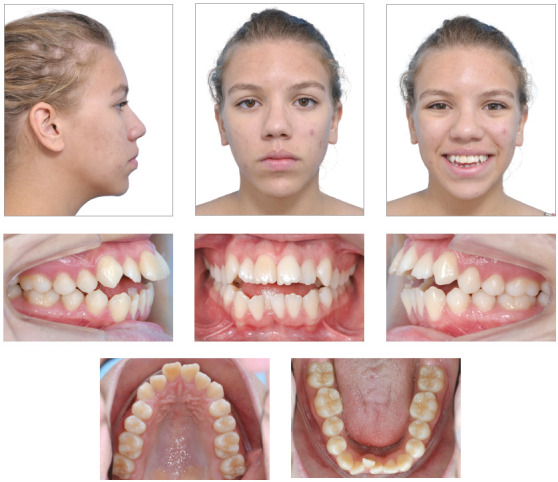
Initial facial and intraoral photographs.

**Figure 2:**
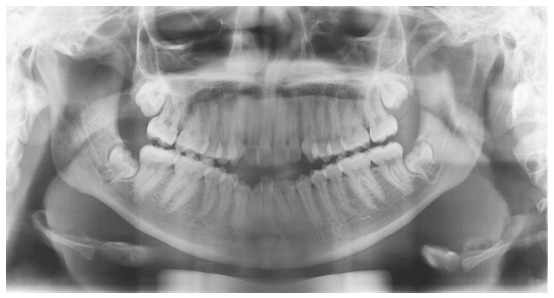
Initial panoramic radiograph.

The patient, a Caucasian woman aged 16 years and 7 months, presented in a private office for initial orthodontic consultation. Her chief complaint was related to the open bite. The patient reported absence of significant records in her medical history. She had never been orthodontically treated. Clinically, no caries or other dental/periodontal problem was detected, and she presented good oral hygiene. The patient presented convex soft tissue profile, Class I malocclusion, permanent dentition, significant anterior open bite, significant overjet, mamelons in the incisal edges of the maxillary and mandibular incisors, maxillary right central incisor presenting yellowish hue, and moderate dental crowding in both dental arches (Figs 1 and 2).

**Figure 3:**
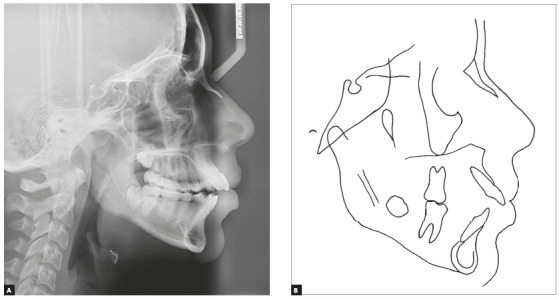
Initial cephalometric radiograph (A) and cephalometric tracing (B).

The skeletal cephalometric assessment showed Class II tendency (ANB = 4°) and hyperdivergent facial type (SN.GoGn = 41° and FMA = 33°), as shown in Table 1 and Figure 3. Cervical vertebrae maturation stage^41^ was CS6, suggesting that her active growth was virtually completed. Her convex profile and hyperdivergent facial features called attention for possible overeruption of molars and detrimental backward (clockwise) mandibular rotation.

**Table 1:** Cephalometric measurements, comparing initial (A) and final (B) lateral radiographs.

	Measurement		Normal	A	B	A/B diff.
Skeletal pattern	SNA	(Steiner)	82°	81°	81°	0
	SNB	(Steiner)	80°	77°	77°	0
	ANB	(Steiner)	2°	4°	4°	0
	Wits	(Jacobson)	♀ 0 ± 2mm	0mm	1mm	1
			♂ 1 ± 2mm			
	Angle of Convexity	(Downs)	0°	7°	8°	1
	Y-Axis	(Downs)	59°	63°	63°	0
	Facial Angle	(Downs)	87°	84°	85°	1
	SN.GoGn	(Steiner)	32°	41°	39°	2
	FMA	(Tweed)	25°	33°	31°	2
Dental pattern	IMPA	(Tweed)	90°	102°	91°	11
	1.NA (graus)	(Steiner)	22°	36°	18°	18
	1-NA (mm)	(Steiner)	4 mm	10mm	3mm	17
	1.NB (graus)	(Steiner)	25°	40°	27°	13
	1-NB (mm)	(Steiner)	4 mm	7mm	3mm	4
	- Interincisal Angle	(Downs)	130°	100°	131°	31
	1 - APg	(Ricketts)	1 mm	6mm	2mm	4
Profile	Upper lip-S line	(Steiner)	0	-2mm	-3mm	1
	Lower lip-S line	(Steiner)	0	4mm	-1mm	5

The patient showed Class I malocclusion; significant overjet (6 mm); anterior open bite (3 mm); permanent dentition with full formed roots and all teeth completely erupted (except third molars, not erupted); moderate dental crowding in the maxillary arch (5 mm) and mandibular arch (5 mm); maxillary and mandibular incisors significantly proclined (except mandibular right central incisor, retroinclined). Maxillary and mandibular arches presented narrow "U" shape. Tongue interposition between maxillary and mandibular dental arches in rest position and tongue thrust during deglutition were detected.

The patient presented leptoprosopic face and convex soft tissue profile; acceptable nasolabial angle and good chin projection; lip sealing, with lips slightly protruded. 

## TREATMENT PLAN AND APPLIED ORTHODONTIC MECHANICS

The treatment objectives were: promote counterclockwise mandibular rotation, to reduce the anterior inferior facial height; increase the chin projection; improve the facial profile, decreasing facial convexity; maintain canines and molars in Class I; achieve adequate overjet and overbite, and correct dental crowding in both dental arches.

Maxillary and mandibular first premolars extractions, and vertical control for molar extrusion during space closure orthodontic mechanics were planned. Intermaxillary elastics would be used when necessary. Orthodontic retention (removable and lower fixed) for at least 12 months after removal of the fixed appliance.

Pre-adjusted brackets and tubes (0.022-in, MBT prescription, American Orthodontics, Sheboygan, WI, USA) were installed in all the teeth, including second molars. Alignment and leveling were achieved with NiTi and stainless steel wires. Extraction spaces closure was performed with 0.017 x 0.025-in stainless steel archwires (upper and lower) with bull loops, and minimal gable bends. Class II elastics (3/16-in heavy) were applied 14 h/day during three months, to differentiate forward movement of mandibular and maxillary molars (mandibular molars having more anchorage loss than maxillary molars). Artistic bends were made in the stainless steel archwires. Inter-maxillary elastics (3/16-in light) were used as needed in the posterior segments, for occlusal settling. 

**Figure 4:**
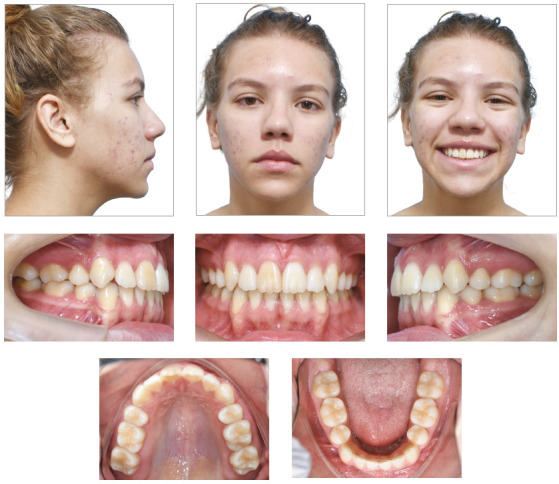
Final facial and intraoral photographs.

Retainers were installed no later than three weeks after fixed appliance removal. Check-up for occlusal relationships (and possible adjustment of occlusal interferences) was made no later than four weeks after retainers had been installed (Fig 4). For retention, a 0.75-mm Essix (Dentsply Raintree, New Orleans, LA) was installed in the maxillary arch, and a 1.0-mm Essix was installed in the mandibular arch. In the mandibular arch, a 0.018-in multistrand wire was also bonded to the canines only, as an adjunct fixed retainer. The patient was instructed to wear the removable retainers for 22 hours/day (except for than meals) for at least 12 months.

## TREATMENT RESULTS

**Figure 5:**
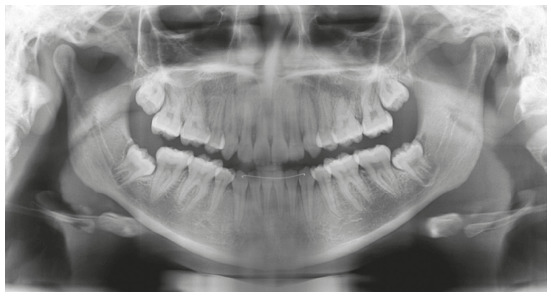
Final panoramic radiograph.

Class I was maintained, and anterior open bite and overjet were corrected, with significant uprighting of the maxillary and mandibular incisors (1.SN difference = 18˚; 1-NA difference = 7 mm; 1.MP difference = 11˚ and 1-NB difference = 4 mm). Furthermore, correct relationship among maxillary and mandibular incisors was achieved. Dental crowding, dental rotations and unlevelled margin ridges were corrected (Figs 4 and 5).

Vertical change of the maxillary incisors was mainly due to the orthodontic mechanic. Two extra millimeters were left forecasting some grinding of incisor mamelons. Change in the position of the maxillary molars, without extrusion, was mainly due to the controlled space closure mechanics.

The maxillary intermolar distance was maintained, and slight decrease occurred in the mandibular one (1 mm). Maxillary and mandibular intercanines distances were minimally increased (1 mm).

**Figure 6:**
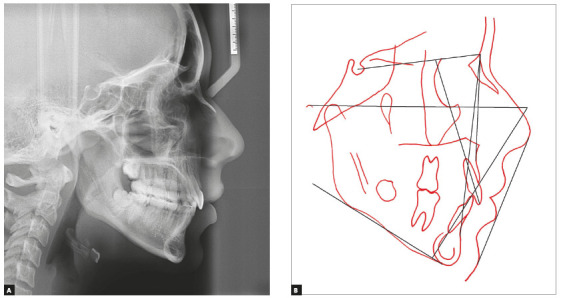
Final cephalometric radiograph (A) and cephalometric tracing (B).

**Figure 7:**
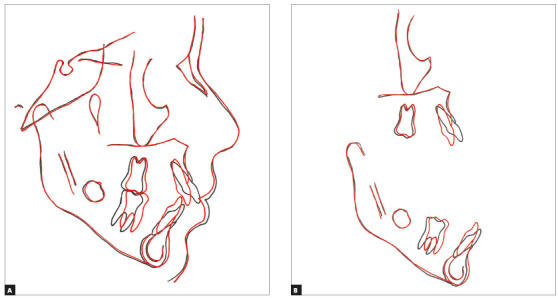
Total (A) and partial (B) superimpositions of initial (black) and final (red) cephalometric tracings.

The facial profile did not change significantly, but there was a slight decrease in the facial convexity and the lip sealing was maintained. Moreover, a slight decrease in the lower anterior facial height and some slight anterior chin projection were due to anterior mandibular rotation (Fig 6). The total and partial superimpositions show minimal reminiscent facial growth, including dentoalveolar changes (Fig 7). Small skeletal changes occurred, other than the significant reduction of the incisors anterior projection.

## DISCUSSION

For subjects presenting facial hyperdivergence, mandibular posture is an important etiologic factor involved.^1^ ^,^ ^42^ During active craniofacial growth, postural deviations can be improved by neuromuscular re-education, and this is the core concept of the application of orthopedic appliances. Therefore, at least hypothetically, in patients with good facial growth potential, counterclockwise mandibular rotation can partially improve the initial hyperdivergent scenario. But in adult patients, strictly speaking, there are just two possible therapeutic alternatives: 1) dental extractions as a method of camouflage or, 2) an orthodontic-surgical approach.

Premolar extractions can improve lip and dental protrusion.^43^ And this happened in the current case, since the patient's final facial profile has become very pleasant. Such effect is contradictory to the common sense that extractions damage facial profiles. When well indicated, extractions can definitely improve facial harmony.^44^

In this current case, all the treatment objectives were successfully achieved after four first premolar extractions: Class I was maintained in the molars, and fully accomplished in the canines; overjet, anterior open bite, and dental crowding were corrected; tongue trust was eliminated, and facial profile convexity was slightly reduced. The final overbite was planned to allow long-term incisal mamelons wear (final overbite of 4 mm, considering that 2 mm - 1 mm of upper incisors and 1 mm of lower incisors - will be ground at a constant and steady pace, with the prospective incisors occlusal function).

Mechanically, when premolar extraction sites are orthodontically closed by *en masse* movements, two basic effects are expected: 1) loss of anchorage of the molars, unless prevented by anchorage methods, and 2) loss of anterior vertical dimension, due to direct or indirect extrusion of the maxillary and mandibular anterior teeth. Such loss of vertical dimension was prevented by gable bends incorporated in the used archwires. However, in open bite cases, such loss of vertical dimension is welcome exactly because it closes the bite. With minimal or no gable effect in the archwires, the open bite was corrected. Passive tongue interposition between maxillary and mandibular incisors and tongue thrust, that in open bite cases are drawbacks, are eliminated when the relationship among maxillary and mandibular incisors is correct. However, achieved results must be monitored to avoid open bite relapse.

**Figure 8:**
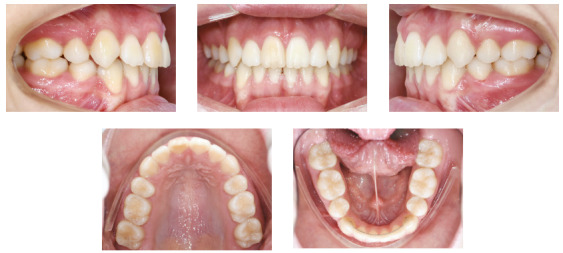
Intraoral photographs, 2 years after completion of fixed orthodontic treatment.

In practice, the risk of relapse in this case is minimal, if any: first of all, good occlusion was obtained (and there is a tendency to be maintained); secondly, the initial muscular pattern, in special of the tongue, was re-established; lastly, because the patient shows great compliance with the wear of removable retainers. A minimal occlusal adjustment was performed six months after debonding. Such fine-tuning is essential to maintain the balance of the occlusion. Two-year follow-up photographs show good stability (Fig 8).

In children and adolescents, anterior open bites with tongue thrust can be treated by fixed or removable appliances, with or without lingual spurs and cribs.^45^ ^-^ ^47^ However, anterior open bites in adult patients are considered skeletal, since the positioning of the anterior teeth implies in permanently deformed dentoalveolar bases and, most of the time, malocclusion is treated with fixed orthodontic appliances and intermaxillary elastics.^48^

Indeed, an orthodontic-surgical approach, with mandibular advancement and counterclockwise rotation of the occlusal plane, can be an alternative therapeutic plan for these cases^49^ But orthognathic surgeries involve extra costs and risks, and provide no full guarantee of long-term stability. Some professionals would claim that orthognathic surgery is the primary option for patients with hyperdivergent retrognathic phenotype, being the premolar extractions option an alternative treatment plan. However, the author of the present report believes the opposite: The premolar extractions choice is the first therapeutic option for young adult patients, mostly teenagers, been orthognathic surgery reserved for selected cases.

The American Board of Orthodontics Discrepancy Index (ABO-DI)^50^ was 39, being this case considered severe mainly because of the hyperdivergent facial phenotype, the presented open bite, and dental crowding. The American Board of Orthodontics Cast-Radiograph Evaluation,^51^ when applied on the final records, scored 3. Therefore, it has been considered that the orthodontic treatment was well succeeded. 

## CONCLUSION

The first premolar extractions therapeutic approach is valid and may be considered the main treatment option for young adult patients presenting hyperdivergent retrognathic phenotype.
